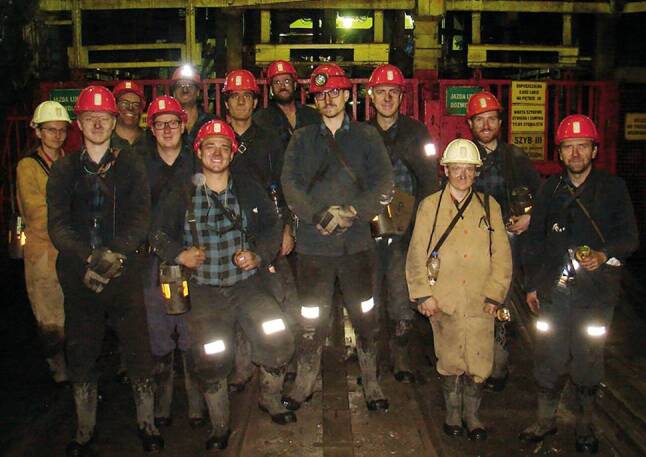# Universitätslehrgang Rock Engineering for Deep Mines

**DOI:** 10.1007/s00501-021-01077-3

**Published:** 2021-01-28

**Authors:** Nikolaus A. Sifferlinger, Horst Wagner, Tobias Ladinig, Birgit Knoll, Hanno Bertignoll

**Affiliations:** Lehrstuhl für Bergbaukunde, Bergtechnik und Bergwirtschaft, Montanuniversität Leoben, Franz-Josef-Straße 18, 8700 Leoben, Österreich

**Keywords:** Rohstoffsektor, Hochschulbildung, Internationalisierung, Lehre, Gebirgsmechanik, Bildungsbedarf, EIT, Innovation, Bewusstseinsbildung, Rohstoffe, Ressourcen, Nachhaltigkeit, Weiterbildung, Wissenschaft, Industrie, Raw Materials, Higher Education, Internationalization, Teaching, Rock mechanics, EIT, Innovation, Resources, Sustainability, Science, Industry

## Abstract

Unter der Leitung des Lehrstuhls für Bergbaukunde, Bergtechnik und Bergwirtschaft der Montanuniversität Leoben wurde ein zweijähriger Berufsausbildungskurs „Rock Engineering for Deep Mines“ (SafeDeepMining) konzipiert. Dieser zielte darauf ab, den Mangel an hochqualifiziertem Gebirgsmechanik-Experten für tiefe Bergwerke zu beheben, mit dem man die Gesteinsdruckprobleme, die den Abbau tiefer Mineralvorkommen gefährden, effektiv angehen kann. Das erklärte Ziel von „SafeDeepMining“ ist es, Bergbauingenieure, Behördenmitarbeiter und Personal in der Beratung und an Universitäten auf dem neuesten Stand der Technik im Felsbau auszubilden, um die europäische Bergbauindustrie bei der Bewältigung von Gebirgsdruckgefahren zu unterstützen, die ihre zukünftigen Untertagebetriebe bedrohen.

## Abstract (en)

A two-year vocational training course “Rock Engineering for Deep Mines” (SafeDeepMining) was designed under the auspices of the Chair of Mining Engineering and Mineral Economics at the Montanuniversität Leoben. It is aimed at remedying the lack of highly qualified rock mechanics experts for deep mines, who will be possible to effectively tackle the rock pressure problems that endanger the mining of deep mineral deposits. The stated objective of “SafeDeepMining” is to provide state-of-the-art training in rock mechanics to mining engineers, government officials and consulting and university personnel to assist the European mining industry in managing rock pressure hazards that threaten its future underground operations.

## Hintergrund des Projektes

Die Stabilität des Gesteins ist eine der größten Herausforderungen im europäischen Untertage-Bergbau, da die Industrie zunehmend dazu übergeht, Erze in größerer Teufe abzubauen. Die Erfahrung zeigt, dass trotz der Fortschritte in der Gebirgsmechanik und numerischer Modellierung immer noch ein erhebliches Defizit bei der Anwendung gesteinsmechanischer Prinzipien in der Bergbauplanung und im Bergbaubetrieb besteht.

Es gibt zahlreiche Beispiele für Probleme im Zusammenhang mit dem Gebirgsdruck, die mit einer guten Gebirgsmechanik hätten vermieden werden können. Allein im Jahr 2019 und 2020 haben eine Reihe von seismischen Ereignissen in tiefen Bergwerken in Europa und darüber hinaus Infrastruktur beschädigt und leider auch Todesopfer gefordert.

In den letzten 50 Jahren wurde die Mehrzahl der staatlich kontrollierten Bergbauunternehmen geschlossen, und viele der führenden Bergbauforschungsorganisationen wurden entweder stillgelegt oder ihre Aktivitäten wurden stark eingeschränkt. Viele Bergbauabteilungen und -programme an europäischen Universitäten sind geschlossen worden. Dies hat zum Verlust von qualifiziertem und erfahrenem Industrie- und Universitätspersonal auf dem Gebiet der Gebirgsmechanik geführt.

Es gab auch einen Verlust an Fachwissen und Infrastruktur in der Bergbauindustrie, bei Regierungsbehörden, die sich mit der Überwachung der Bergbauaktivitäten befassen, bei Forschungsorganisationen und Universitäten. All dies bedeutet, dass neue Entwicklungen in der Gebirgsmechanik nicht oder nur unvollständig angewandt werden.

## Erste Erfahrungen

Im Jahr 2018 wurde unter der Leitung des Lehrstuhls für Bergbaukunde, Bergtechnik und Bergwirtschaft der Montanuniversität Leoben mit der Gestaltung eines zweijährigen Ausbildungskurses „Rock Engineering for Deep Mines“ (SafeDeepMining) begonnen. Unter der fachlichen Leitung von Prof. Horst Wagner wurden mit den Universitäten TU Bergakademie Freiberg, TU Clausthal, University of Pretoria und Silesian University of Technology sowie KGHM Cuprum, ZAMG und GEODATA kompetente Partner an Bord geholt. Es entstand ein Universitätslehrgang mit Sitz an der Montanuniversität Leoben.

Von 2018 bis 2020 wurde dieser Universitätslehrgang mit dem Titel „Rock Engineering for Deep Mines“ von EIT RawMaterials gefördert und zielte darauf ab, den Mangel an hochqualifizierten Gebirgsmechanik-Experten für tiefe Bergwerke zu beheben, mit dem man die Gesteinsdruckprobleme, die den Abbau tiefer Mineralvorkommen gefährden, effektiv angehen kann. Das erklärte Ziel von „SafeDeepMining“ ist es, Bergbauingenieure, Behördenmitarbeiter und Personal in der Beratung und an Universitäten auf dem neuesten Stand der Technik im Felsbau auszubilden, um die europäische Bergbauindustrie bei der Bewältigung von Gebirgsdruckgefahren zu unterstützen, die ihre zukünftigen Untertagebetriebe bedrohen. Aus diesem Grund haben Bergbauunternehmen wie WOLFRAM, RHI Magnesita und KGHM Cuprum dieses Programm ausgewählt, um ihre Experten weiterzubilden.

Das Programm engagierte die Industrie im Jahr 2018/2019 (EUROMINES, polnische Bergbauindustrie, LKAB, RHI Magnesita, österreichische Bergbauindustrie, deutsche Bergbauindustrie, New Boliden, European Federation of Geologists) und das Feedback war erneut positiv, mit der Bitte, die Programmmodule von zwei auf eine Woche zu verkürzen. Dies wurde getan, und auch der Inhalt des Ausbildungsprogramms wurde an den Input der Bergbauindustrie angepasst.

Der Pilotkurs begann im Oktober 2019 und wird voraussichtlich im März 2021 beendet. Die ersten zwölf Teilnehmer werden im Frühjahr 2021 den Lehrgang abschließen.

Die COVID 19-Beschränkungen erforderten eine Umplanung und Digitalisierung des Lehrgangs.

**Figure 1:**
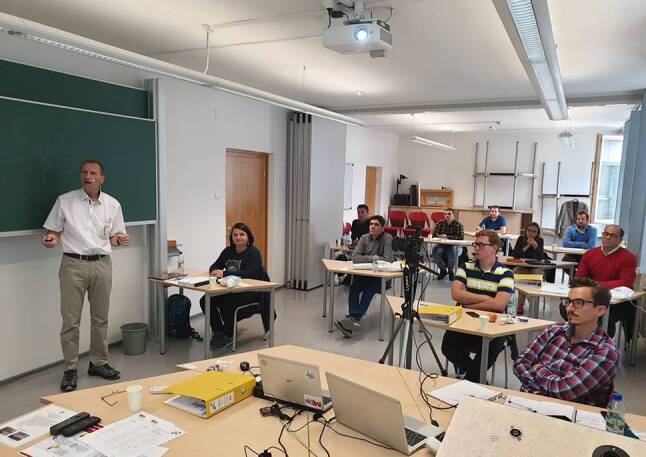

Im Herbst 2020 wurde daher das sechste Modul des Lehrgangs an der Montanuniversität in hybrider Form abgehalten. Jene Kursteilnehmer, die zu diesem Zeitpunkt noch reisen konnten, waren in Leoben präsent, die anderen waren mittels eines Live-streams online dabei. (Abb. 1).

Mit den gewonnenen Erkenntnissen und dem Feedback der Industrie und der Interessenvertreter wurde zusätzlich zum Universitätslehrgang der Kurs zu einem vierwöchigen Trainingsprogramm komprimiert, ebenso wird die Teilnahme auch in einzelnen Modulen angeboten.

Es stehen damit drei Varianten des Ausbildungsprogrammes zur Verfügung:

1. Ein zweijähriger Universitätslehrgang „Rock Engineering for Deep Mines“ mit 60 ECTS (Laufzeit 2019–2021 und 2021–2023)
2. Ein vierwöchiger Kurs mit ausgewählten Kapiteln des Universitätslehrganges (Beginn 2021)
3. Einzelne einwöchige Ausbildungsmodule (11 bestehende Module)

Inhalte:

- Gebirgsmechanik und Messtechnik;
- Gebirgsschläge;
- Seismische Effekte durch Bergbauaktivitäten;
- Gebirgsmechanisches Risikomanagement;
- Ausbaudimensionierung

Ausbildungspartner:

Montanuniversität Leoben, TU Clausthal, Technische Universität Gliwice, TU Bergakademie Freiberg, University of Pretoria, GEODATA Group, KGHM Cuprum Ltd, Wolfram Bergbau und Hütten AG, ZAMG.

**Figure 2:**
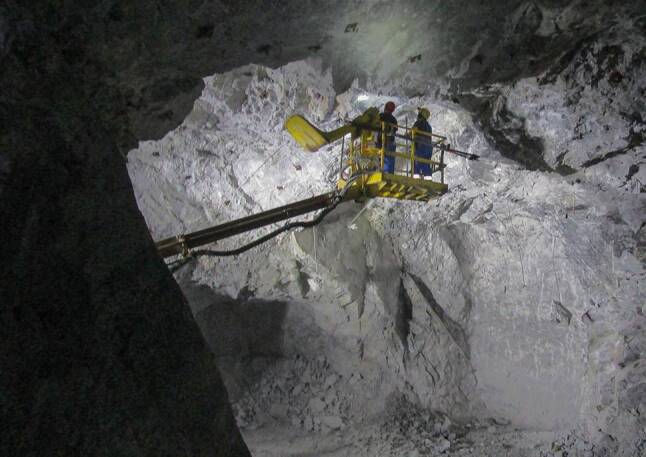

Einen wesentlichen Schwerpunkt der Ausbildung bildet der Erfahrungsaustausch der Teilnehmer untereinander und die Befahrung betroffener Bergwerksunternehmen, wie z. B. die Bergbaue in Mittersill und in der Breitenau oder das Polnische Steinkohlebergwerk Budryk (Abb. 2 und 3).

**Figure 3:**